# Editorial: Have We Got Better in Making Our Schizophrenia Patients Better?

**DOI:** 10.3389/fpsyt.2020.618417

**Published:** 2020-11-27

**Authors:** Erika Jääskeläinen, Jouko Miettunen, Anthony O. Ahmed

**Affiliations:** ^1^Center for Life Course Health Research, University of Oulu, Oulu, Finland; ^2^Medical Research Center Oulu, Oulu University Hospital and University of Oulu, Oulu, Finland; ^3^Department of Psychiatry, University Hospital of Oulu, Oulu, Finland; ^4^Department of Psychiatry, Weill Cornell Medicine, Cornell University White Plains, New York, NY, United States

**Keywords:** schizophrenia, recovery, intervention, cognition, psychosis

Despite its heterogeneity in prognosis, schizophrenia is considered as a disorder with relatively poor outcomes. For some individuals, schizophrenia increases the risk for suicide, psychiatric and medical comorbidities, recurrent relapses, treatment resistance, and poor functioning. Remission and recovery are however well-documented, and ~13.5% of individuals with schizophrenia experience good social and clinical recovery (1).

In this Research Topic, we wanted to find answer to the question *Have we got better in making our schizophrenia patients better?* This collection includes 12 articles on recovery and psychosocial interventions, early intervention and prevention services and cognition in psychoses.

In a study with hospitalized patients with schizophrenia or schizoaffective disorder, Chen et al. found that resilience and positive coping mediated the relationship between negative symptoms and functional disability. Their results suggest that resilience and positive coping are important treatment targets for attenuating the impact of negative symptoms. In a study by Chien et al., along with mindfulness based psychoeducation intervention, two facets of mindfulness, “observing” and “acting with awareness,” were related to positive outcomes of recent onset psychosis. This study was first to show mechanism explaining the benefits of mindfulness skill on psychoses. Kim et al. identified an association between depression and functional mobility in schizophrenia. Improving functional mobility by treating depression may have considerable therapeutic value when aiming to functional recovery.

The last few decades have seen an increased interest in early interventions for patients with psychotic illnesses or those with clinical high risk for psychosis (CHR-P). Fusar-Poli et al. describe the Pan-London Network for Psychosis-Prevention (PNP), including four different early intervention teams, with heterogenous characteristics of the catchment areas and service users. Psychosis transition rate (30% in 4-year follow-up) in PNP suggests that the actual transition risk of clinical cohorts may not be declining, as opposed to research cohorts. Standalone teams were more established and successful than teams that share their resources with other mental health services. Fusar-Poli et al. paper highlights crucial operational issues which need careful consideration in the future planning of CHR-P services. Berze et al. present a study protocol of a 6 months early intervention program in Latvia—the Latvian Early Intervention Program—for first-episode patients in a low-resourced outpatient service. The success of early intervention and prevention depends on efficient detection on individuals in need of assessment and care, and this depends on help-seeking behavior of the patients and families. Wong et al. made qualitative study on caregivers. They found that high level of right knowledge on schizophrenia and its treatment and low level of stigma related to better professional help-seeking behavior during first episode schizophrenia.

Cognitive deficits have been heavily studied in schizophrenia. To date, the majority of clinical trials of medications developed to address cognitive deficits in schizophrenia have been unsuccessful (2, 3). Three articles in the collection focused on cognition. In their review, Cotter et al. suggested that one of the reasons pharmacological trials of cognition have been unsuccessful may be because few studies have actually screened for patients with significant cognitive deficits and may have therefore included several cognitively “normal” patients. They found that only 11.5% of trials had included cognitive function as an eligibility criterion. Moreover, none examined the moderating effect of cognitive performance. Therefore, whether patients with more impaired cognition gain more from cognitive enhancing drugs remains an empirical question. Szczepanowski et al. showed that metacognitive accuracy in self-monitoring tasks in patients with schizophrenia can be improved by engaging a method of assessment that imposes a cost on overconfident judgments of incorrect responses. By implication, their study demonstrates that inducing an aversion to loss of desired incentives could increase metacognitive accuracy in self-monitoring task by decreasing over-confidence and perhaps other cognitive biases. Adamowicz et al. found that the dietary intervention addressing metabolic dysfunction improved cognitive performance whereas cognitive abilities remained unchanged in the non-dietary intervention group.

High rates of medication non-adherence and overall treatment disengagement remain a challenge in the care of people with psychotic illnesses. Studies have reported medication non-adherence rates that frequently exceed 30%, including rates as high as 50% in first-episode psychosis patients (4, 5). In this collection, Mucci et al. make the case about the importance of treatment alliance and the need to understand its nature and factors that may enhance or impede alliance in treatment. They present psychoeducation and shared-decision making as interventions that can foster alliance with patients and caregivers. Shared-decision making goes beyond psychoeducation as it involves a bidirectional exchange of information between two experts—the practitioner with knowledge of psychiatric care, and the patient with knowledge of their own history, preferences, and personal goals (6).

Like shared decision-making, motivational interviewing has been examined as an intervention to enhance treatment adherence. Motivational interviewing is a style of communication that targets patients' own decisional conflicts and ambivalence about improvement through “change talk” to enhance their commitment. Dobber et al. examined the active ingredients of motivational interviewing including clinician factors, client factors, and mechanisms of change. They wanted to identify what ingredients may trigger mechanisms of change. They found that clinician factors, in particular, reflection and questions about medication adherence that subsequently lead to change talk on the part of the patient triggered mechanisms of change. These variables, underscored in conjunction with other motivational interviewing principles including trust and empathy, allow meaningful conversations about the role of medication adherence in the patient's own self-identified values and long-term goals.

The goal in the treatment of schizophrenia is recovery. But what does it mean? Is it defined by the clinical, society, or the patient him/herself? Most of the studies on recovery in schizophrenia have focused on clinician's assessment of recovery. However, patients and advocates of the recovery movement that started about four decades ago have highlighted the importance of “personal recovery,” the idea that people with schizophrenia can live a productive and satisfying life, despite the limitations of illness. The patient-based definition of personal recovery indicates a development of new meaning and purpose in one's life, due to growing beyond the catastrophic effects of mental illness (7).

In this collection Lee et al. describe the construct of post-traumatic growth (PTG) in psychosis. Drawing on personal experience, theoretical developments, and extant research, the authors argue that deeper insights into how mental health professionals can support their patients to achieve PTG could bring existing mental healthcare services to greater heights.

From a recovery-oriented perspective, a more appropriate question would be *Have we got better in helping our patients better?* Recovery is not about clinicians doing something (making better) for patients to make them recover clinically. Rather the role of the clinician is to deploy evidence-based interventions to the service of the patient's personal goals and aspirations. In this role, the clinician adopts a patient-centered approach to treatment, engages in information sharing for patients and families and shared decision-making, builds trust, and works to decrease stigma.

During last decades, several new options for psychosocial care and rehabilitation have been developed (8). We have gone far from the early times of solely institutional care or solely drug treatment. The patient-centered, recovery-oriented care has raised to be used in combination of evidence-based pharmacological and psychosocial treatment. We do believe that we clinicians and scientists have got better in helping our patients better.

## Author Contributions

EJ wrote the first draft of the manuscript. AA and JM provided critical revision and important intellectual contributions to the manuscript. All authors have read and approved the submitted version.

## Conflict of Interest

The authors declare that the research was conducted in the absence of any commercial or financial relationships that could be construed as a potential conflict of interest.
